# Pattern and trends of *Helicobacter pylori* genotypes in gastric cancer: A Kenyan 8-year study

**DOI:** 10.3389/fmed.2023.1119513

**Published:** 2023-02-17

**Authors:** Priscilla Njenga, Allan Njau, Zahir Moloo, Gunturu Revathi, Evariste Tshibangu, Yoshio Yamaoka

**Affiliations:** ^1^Department of Pathology, The Aga Khan University Hospital, Nairobi, Kenya; ^2^Department of Environmental and Preventive Medicine, Oita University, Ōita, Japan

**Keywords:** *H. pylori* (*Helicobacter pylori*), genotype, gastric cancer, Kenya, trends

## Abstract

**Background:**

Notable geographic and temporal variations in the prevalence and genotypes of *Helicobacter pylori*, in relation to gastric pathologies, have been observed; however, their significance and trends in African populations is scarcely described. The aim of this study, was to investigate the association of *H. pylori* and its respective *CagA* and vacuolating cytotoxin A (*VacA*) genotypes with gastric adenocarcinoma, and to describe the trends of *H. pylori* genotypes over an 8-year period (2012–2019).

**Materials and methods:**

A total of 286 samples of gastric cancer cases and benign controls (one-to-one matching), from three main cities in Kenya, between 2012 and 2019 were included. Histologic evaluation, and *CagA* and *VacA* genotyping using PCR, was performed. Distribution of *H. pylori* genotypes was presented in proportions. To determine association, a univariate analysis was conducted using a Wilcoxon rank sum test for continuous variables, and a Chi-squared test or Fisher’s exact test for categorical data.

**Results:**

The *VacA s1m1* genotype was associated with gastric adenocarcinoma, {odds ratio (OR) = 2.68 [confidence interval (CI) of 95%: 0.83–8.65]; *p* = 0.108}, whilst *VacA s2m2* was associated with a reduced probability of gastric adenocarcinoma [OR = 0.23 (CI 95%: 0.07–0.78); *p* = 0.031]. No association between cytotoxin associated gene A (*CagA*) and gastric adenocarcinoma was observed.

**Conclusion:**

Over the study period, an increase in all genotypes of *H. pylori* was seen, and although no predominant genotype was noted, there was significant year-to-year variation, with *VacA s1* and *VacA s2* showing the greatest variation. *VacA s1m1* and *VacA s2m2* were associated with increased, and reduced risk of gastric cancer, respectively. Intestinal metaplasia and atrophic gastritis did not appear to be significant in this population.

## Introduction

*Helicobacter pylori* is likely the most genetically diverse bacterial species and the most prevalent human pathogen (1, 2). This organism colonizes the stomach of approximately half of the world’s population, and is etiologically associated with a wide spectrum of diseases, ranging from chronic gastritis, to peptic ulcer disease, and gastric adenocarcinoma (2, 3).

A recent systematic review and meta-analysis on the global prevalence of *H. pylori* infection, found that Africa had the highest pooled prevalence (70.1%) whilst Oceania and North America had the lowest (24.4%) (2). This study included works published from 62 countries between 1970 and 2016, using heterogeneous modalities of detection, including, serology, stool antigen test, urea breath test, culture and histopathology. Locally, a Kenyan study sampling 487 patients with dyspepsia, and utilizing rapid urease, histopathology and culture tests, found the prevalence to be 40.86%, lower in comparison to reports from other developing countries (4).

The GLOBOCAN 2020 report, listed gastric cancer as the fifth most common cancer worldwide, with over one million new cases reported, more than 70% of these found in developing countries. In addition to this, it was found to be the fourth leading cause of cancer-related mortality worldwide, in both sexes, accounting for nearly 769,000 deaths (i.e., 7.7% of all new cancer related deaths) (5); and interestingly, African patients were found to present not only at a younger age (between the third and the fourth decade), but also a more advanced stage of disease (6).

The majority of gastric cancers are sporadic, with chronic *H. pylori* infection found to be the main risk factor in up to 90% of gastric cancer cases (5). Studies have shown a sixfold increase in the risk of gastric cancer among *H. pylori* infected populations, in comparison to the uninfected (1), and as such, it has been classified as a type 1 carcinogen by the International Agency for Research on Cancer (IARC), WHO (7).

The fact that only a subset of infected people develop severe gastrointestinal disease has been attributed to several factors related to the host, environment and bacterium (3). With reference to the bacterium, cytotoxin associated gene A (*CagA*) and the vacuolating cytotoxin A (*VacA*), appear to be the major virulence factors involved in disease pathogenicity, in an allele-dependent manner, potentially explaining the global geographic distribution of gastric adenocarcinoma (8). The meta-analysis by Pormohammad et al. determined that these molecules are significantly associated with an increased risk of gastric cancer, with an odds ratio (OR) to detect of 2.82 and 1.75 for *CagA* and *VacA*, respectively. Moreover, the prevalence of the *CagA* gene in gastric adenocarcinoma was found to be 74%, with the *VacA s1m1* mosaic combination identified in 52.4% of cases (9). A local study investigating the association between *H. pylori* and clinical outcomes, found that *H. pylori* was detectable in 62.9% of 127 patients with dyspepsia. Of the 127 patients, only 10 had a diagnosis of gastric cancer. The prevalence of the *CagA* gene was found to be 48.75%, with no significant association with gastrointestinal disease, including cancer, and in contrast, the *m2*, *i2*, and *s2* alleles of the *VacA* gene were found in 65, 52, and 49%, respectively, with varying but significant associations with gastric cancer (10).

Despite having the highest pooled prevalence of *H. pylori*, the rates of gastric cancer in Africa remain among the lowest worldwide. In Eastern Africa for example, the rates are 4.9 and 4.2 per 100,000 in men and women, respectively, whilst in Eastern Asia, the rates are 32.5 and 13.2 per 100,000 in men and women, respectively (5). In 2020, the incidence of gastric cancer in Kenya was 7.2 per 100,000 in the male population, and 7.7 per 100,000 in the female population, with a mortality rate of more than 6.6 per 100,000 (5). The prevalence in Nairobi, Kenya, according to the only available data from a population-based cancer registry between 2004 and 2008, was 6.2% (11); however, it is believed that this may be higher given the GLOBOCAN estimated incidence rates for the Eastern Africa region. Different tumor biology, and perhaps an overestimation of the *H. pylori* prevalence may confound the actual situation (2, 6); however, due to a paucity of data, particularly in African populations, this cannot be commented on.

Cancer continues to show an upward trend, more so in developing countries. As pertains specifically to gastric adenocarcinoma, the knowledge of the distribution and significance of *H. pylori* is not only scant, but remains inadequately investigated in African populations, particularly in Kenya. Furthermore, the *H. pylori* genotypes not only vary from one region to the next, but also show variation over time. As such, it is necessary to obtain more up-to-date data within any given population, with the aim of expanding the knowledge of the role, and magnitude this bacterium plays in the gastric cancer burden (2). Ultimately, stratification of persons at increased risk of developing gastric cancer, and management strategies that involve *H. pylori* eradication programs and follow-up, can be rationalized based upon the findings of such studies.

Thus, the objectives of this study are to describe the association of *H. pylori* and its respective *CagA* and *VacA* cytotoxic genes with gastric adenocarcinoma and, to describe the trends of *H. pylori* genotypes over the last 8 years (2012–2019) in Kenya.

## Materials and methods

### Study design and setting

A case-control laboratory-based study was designed including three study sites; The Aga Khan University Hospital Nairobi (AKUHN), and The Aga Khan Hospitals (AKH) Mombasa, and Kisumu. These institutions are located in the three main cities in Kenya, and represent cosmopolitan populations.

### Sample size and sampling process

Expecting that the age and the sex group might be determinant for both the histological lesion and the presence of *H. pylori*, consecutive samples of gastric cancer cases reported between 2012 and 2019 were matched randomly from a pool of benign gastric biopsies in a one-to-one process. With a minimum OR to detect of two (based on the meta-analysis by Pormohammad et al.), and a projected power of greater than 80%, the expected minimum number of cases was 143, combined from all three facilities, with an equal number of controls and hence a total sample size of 286. All samples were de-identified formalin fixed paraffin embedded (FFPE) tissue blocks, and, were either biopsy, excision or gastrectomy specimens. Samples with insufficient material were excluded, whilst only those with biodata (age and sex) were included.

### Laboratory procedures

#### Sectioning of FFPE blocks

Selected FFPE blocks were sectioned for both histological assessment and molecular analysis of *H. pylori*, *VacA* and *CagA* genes. The first sections were cut at 3 μm for standard haematoxylin and eosin (H&E) staining. This was followed by five sections of 6–8 μm each, placed in 2 ml micro centrifuge tubes for DNA extraction. To avoid carry-over contamination, a new blade was used for each FFPE block, and the microtome overlay cleaned after each case with DNA cleaner. As a quality control measure to monitor effectiveness of carry-over contamination, prevention procedures were conducted using a blank paraffin block sectioned after every 10 cases and processed for PCR alongside the other cases.

#### Histology

Standard H&E staining was performed on 3 μm sections of all FFPE blocks of the cases and controls on the Dako autostainer platform (Dako, Denmark). Joint histologic review of all the cases and controls using a consensus approach by two registered pathologists and one resident was performed. Gastric cancer was classified using the Lauren classification, while gastritis using the Sydney system. The presence of *H. pylori* was assessed on newly stained H&E and original Giemsa sections, and was reported as positive if identified on either stain.

##### Isolation and purification of DNA

Total genomic DNA was extracted from the tissue sections as per the manufacturer’s protocol using a commercial kit (QIAamp DNA FFPE tissue kit, Qiagen, Hilden, Germany). Following dewaxing, which involved two washes in xylene and two washes in absolute ethanol, the tissue pellets were suspended in 180 μL of ATL buffer and 20 μL of proteinase K. These were incubated at 56°C until completely lysed, and then at 90°C for 1 h, followed by addition of 200 μL of AL buffer and 200 μL of ethanol (99.5%), prior to purification through QIAamp spin columns. Extracted DNA was concentrated using DNA Clean & Concentrator-100 (Edge Bio’s Performa^®^ DTR Gel Filtration Cartridges, San Jose, CA, USA) following the manufacturer’s protocol.

##### *CagA* and *VacA* genotyping

Amplification and genotyping of the *Cag* and *Vac A*, *s*, and *m* regions was performed using end-point PCR, with previously published PCR conditions from similar studies referred to (12). To confirm the presence of *CagA* gene, we corrected a set of nucleotide primers previously described (13), based on sequence variations noted in the Kenyan *H. pylori* isolates (14), as well as other African isolates retrieved from.^1^ This process was performed using the Qiagen CLC genomics workbench, and the following forward and reverse primers selected: *CagA*-CA-OMF: 5′-CAA GCA AAA AGC GAC CTT GAA A-3′ and *CagA*-Ke-OMR: 5′-ACA CCA TTC TTA ACG GAT TG-3′(248 bp). The primers used for *VacA s_1_/s_2_* were VA1-F 5′- ATGGAAATACAACAAACACAC-3′, and VA1-R 5′-CTGCTTGAATGCGCCAAAC-3′ (product size 259/286 bp); and, the primers used for *VacA m_1_/m_2_* were, VAG-F 5′-CAATCTGTCCAATCAAGCGAG-3′ and VAG-R 5′- GCGTCAAAATAATTCCAAGG-3′ (product size 570/645 bp). The specificity of the primer set was assessed using the Blast search through the NCBI database.^2^ Amplification was achieved by an initial denaturation step of 10 s at 98°C, followed by 30 cycles of 10 s at 98°C, 30 s at 55°C and 20 s at 72°C; and a final extension step of 1 min at 72°C. Each PCR reaction (12.5 μL), contained 6.25 μL premix (Takara Emerald Amp^®^ Max PCR Master mix, Kusatsu, Japan), 2 μL of forward and reverse primers each and 2.25 μL of purified DNA. The PCR products were electrophoresed using Agarose gel (Nippon gene Agarose S, Tokyo, Japan) at 2 g/100 ml, with ethidium bromide concentration of 3 μL/100 ml at a voltage of 135 volts, for 35 min.

##### Reference samples

As reference samples for the assessment of *Vac s* and *m* region, *H. pylori* Tx-30a (ATCC 51932) (*CagPAI* negative, *s_2_m_2_*) and *H. pylori* 26695 (*CagA* positive, *s_1_m_1_*) were cultured and used as positive controls for the experiment. Similarly, for *CagA*, Kenyan isolate 78 (*CagA* positive, *s_1_m_1_*) and *H. pylori* 26695 (*CagA* positive, *s_1_m_1_*). These isolates were inoculated under a biological safety cabinet onto a *Helicobacter* selective Agar medium (Nissui Pharmaceutical co., Ltd., Tokyo, Japan) and incubated for 10 days. The colonies growing on the plates were identified and sub cultured for 3–4 days at 37°C in microaerophilic conditions (10% CO_2_, 5% O_2_, and 85% N_2_). Sub culture was on Brucella Agar plates (Becton Dickinson, Sparks, MD, USA), supplemented with 7% horse serum (Nippon Biotest Laboratories Inc., Tokyo, Japan). The colonies were identified as small, round, translucent and the organisms were gram-negative and positive for the urease test.

## Statistical analyses

Baseline characteristics, histological and molecular data for each sample were entered into a Microsoft Excel database. The results for each of the target genes were recorded in a binary format (i.e., absent or present for both the case and control groups); and compared using a 2 × 2 contingency table. A univariate analysis was conducted using a Wilcoxon rank sum test for continuous variables and a Chi-squared test or Fisher’s exact test for categorical data, when comparing the cases and controls. The homogeneity of variances and normality of distributions were assessed using the Levene and Shapiro–Wilk tests. Odds ratios (ORs) were used to measure the association between qualitative variables and a *p*-value of less than 0.05 was considered statistically significant.

### Ethical considerations

This study was conducted after ethical approval from The Aga Khan University, Institutional Ethics Review Committee (IERC), (reference 2019/REC-40). Under this approval, only de-identified archival tissues were used.

## Results

### Baseline characteristics

Overall, 286 samples comprising 143 gastric cancer cases and an equal number of age, and sex matched controls were analyzed (Supplementary Table 1). The age range was between 21 and 92 years, with the mean and median ages found to be 61 years on both accounts. The highest frequency of age distribution (51.7%) was found to be in patients equal to or greater than 60 years; with samples from patients between the 5 and 6th decades accounting for 37.1% (53/143), and those between 21 and 40 years accounting for 11.2% (16/143). The male to female ratio of gastric cancer in this sample was 1.5:1. For the majority, (53.1%) the location of the cancer was not specified, 8.9% (27/143) were from the gastric antrum and 16.8% (24/143) from the gastroesophageal junction (GEJ), 4.2% each from the body and fundus, and 2.8% from the cardia. The control samples had a nearly similar distribution; however, there were larger numbers from the gastric antrum and body compared to the GEJ. Ultimately, the contribution of samples from AKH Mombasa was too small (1.4%) for any accurate assessment of *H. pylori* genotypes in that city to be conducted (Table 1).

**TABLE 1:** Baseline characteristics of the samples analyzed.

Characteristics	Cases *n* = 143 (%)	Controls *n* = 143 (%)	All samples *n* = 286 (%)
**Age group**			
(21–40)	16 (11.2)	16 (11.2)	32 (11.2)
(41–60)	53 (37.1)	53 (37.1)	106 (37.1)
(61–80)	66 (46.2)	66 (46.2)	132 (46.2)
> 80	8 (5.6)	8 (5.6)	16 (5.6)
**Gender**			
Female	58 (40.6)	58 (40.6)	116 (40.6)
Male	85 (59.4)	85 (59.4)	170 (59.4)
**Study sites**			
AKH_Kisumu	32 (22.4)	32 (22.4)	64 (22.4)
AKH_Mombasa	2 (1.4)	2 (1.4)	4 (1.4)
AKUH_Nairobi	109 (76.2)	109 (76.2)	218 (76.2)
**Type of samples**			
Biopsies	134 (93.7)	143 (100)	277 (96.9)
Excisions	9 (6.3)	0 (0)	9 (3.1)
**Site of sample collection**			
Antrum	27 (18.9)	56 (39.2)	83 (29.0)
Cardia	4 (2.8)	0 (0.0)	4 (1.4)
GEJ	24 (16.8)	1 (0.7)	25 (8.7)
Body	6 (4.2)	23 (16.1)	29 (10.1)
Fundus	6 (4.2)	2 (1.4)	8 (2.8)
Gastric, site not specified	76 (53.1)	61 (42.7)	137 (48)

### Histologic features and *H. pylori* genotypes in gastric adenocarcinoma

**FIGURE 1:**
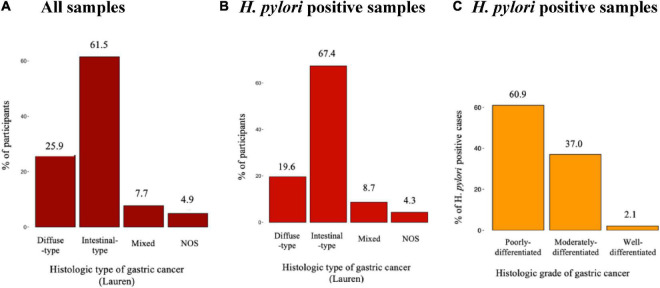
Distribution of *Helicobacter pylori* amongst the histologic types and grades of gastric adenocarcinoma. **(A)** All samples, **(B)** *H. pylori* positive samples, and **(C)** *H. pylori* positive samples.

As depicted in Figure 1 the majority of the gastric adenocarcinoma cases, were of the intestinal type, 61.5% (88/143). The diffuse, mixed and indeterminate types accounted for 25.9% (37/143), 7.7% (11/143), and 4.9% (7/143), respectively. A total of 46 (32.1%) gastric adenocarcinoma cases were found to be positive for *H. pylori* on both molecular and histologic analyses, with the error of double counting circumvented, and 97 (67.8%) were *H. pylori* negative.

Out of the 46 *H. pylori* positive cases, 67.4% (31/46) were of the intestinal type, while the diffuse, mixed and indeterminate types accounted for 19.6, 8.7, and 4.3%, respectively. The majority of the *H. pylori* positive gastric cases were found to be poorly differentiated, 60.9% (28/46), while moderately and well-differentiated cases accounted for 37% (17/46) and 2.2% (1/46), respectively (Figure 1).

Further *H. pylori* genotype analysis was successful in only 38 of the *H. pylori* positive cases. These included 27 cases of the intestinal subtype, eight of the diffuse, and two of the mixed type, with only one indeterminate case. Specific to the intestinal type, the *CagA* genotype was identified in 48.1% (13/27) of the *H. pylori* positive cases, with slightly over a half, 51.9% (14/27), found to be negative. *VacA s1* genotype was identified in 14 cases (51.9%) and *VacA s2* in 11 cases (40.7%), while *VacA m1* and *m2* were encoded in 55.6% (15/27) and 33.3% (9/27) accordingly. The predominant mosaic combination was *VacA s1/m1*, with a frequency of 33.3% (9/27) followed by *s1/m2*, 14.8% (4/27). The other genotypes, *s2/m1* and *s2/m2*, were identified in 5 (18.5%) and 4 (14.8%) cases, respectively. Further to the *H. pylori* positive diffuse-variant cases, 75.0% (6/8) were positive for *CagA*. *s1* and *s2 Vac* regions were encoded in the following frequencies, 87.5% (7/8) and 25.0% (2/8); with *m1* and *m2* found to be present in 75.0% (6/8) and 37.5% (3/8), respectively. The predominant mosaic combination was once again *VacA s1/m1* with a frequency of 62.5% (5/8). *s1/m2* was detected in 2 cases (25.0%), and of the remaining genotype combinations, only *s2/m1* was recorded (11.1%) (Table 2).

**TABLE 2:** *Helicobacter pylori* genotype distribution amongst the histologic subtypes of gastric adenocarcinoma.

*H. pylori* genotype	Intestinal-type *n* = 27 (%)	Diffuse-type *n* = 8 (%)	Mixed *n* = 2 (%)	Indeterminate *n* = 1 (%)
*CagA* positive	13 (48.1)	6 (75.0)	0 (0.0)	0 (0.0)
*VacA s1*	14 (51.9)	7 (87.5)	1 (50.0)	0 (0.0)
*VacA s2*	11 (40.7)	2 (25.0)	1 (50.0)	1 (100.0)
*VacA m1*	15 (55.6)	6 (75.0)	1 (50.0)	0 (0.0)
*VacA m2*	9 (33.3)	3 (37.5)	0 (0.0)	1 (100.0)
*VacA s-m1*	1 (3.7)	0 (0.0)	0 (0.0)	0 (0.0)
*VacA s-m2*	1 (3.7)	0 (0.0)	0 (0.0)	0 (0.0)
*VacA s1m1*	9 (33.3)	5 (62.5)	0 (0.0)	0 (0.0)
*VacA s1m*-	1 (3.7)	0 (0.0)	1 (50.0)	0 (0.0)
*VacA s1m2*	4 (14.8)	2 (25.0)	0 (0.0)	0 (0.0)
*VacA s2m1*	5 (18.5)	1 (12.5)	1 (50.0)	0 (0.0)
*VacA s2m*-	2 (7.4)	0 (0.0)	0 (0.0)	0 (0.0)
*VacA s2m2*	4 (14.8)	0 (0.0)	0 (0.0)	1 (100.0)

The commonest pathology amongst the controls was chronic gastritis, 60.8% (87/143), with activity (neutrophil infiltrate) present in 43.7% of these. Biopsies exhibiting no pathology accounted for 28.7% (41/143), and other forms of gastritis (acute, atrophic and reactive) formed the remainder of the samples. Only 43 of the 143 controls tested positive for *H. pylori*, with the error of double counting circumvented. The majority of these were chronic gastritis (76.7%). Intestinal metaplasia and atrophic gastritis were found to be rare, 0.07% (10/143) and 0.06% (8/143), respectively.

### Virulence and host gene detection in *H. pylori* cases and controls

In this study, the presence of the *VacA s2m2* genotype was significantly associated with a reduced probability of having gastric adenocarcinoma {OR = 0.23 [confidence interval (CI) 95%: 0.07–0.78]; *p* = 0.031}. In contrast, the *VacA s1m1* genotype was associated with gastric adenocarcinoma, however, this association was not statistically significant [OR = 2.68 (CI 95%: 0.83–8.65); *p* = 0.108]. There was no significant association observed between the *CagA* genotype and the gastric adenocarcinoma [OR = 0.69 (CI 95%: 0.25–1.87); *p* = 0.628] (Table 3).

**TABLE 3:** Detection and distribution of *H. pylori* virulence genes in cases and controls.

Genotype		OR (CI 95%)	Cases	Controls	*p*-value
			*n* = 143 (%)	*n* = 143 (%)	
*CagA*	Positive	0.69 (0.25, 1.87)	21 (55.2)	18 (64.3)	0.628
	Negative		17 (44.7)	10 (35.7)	
*VacA m1*	Positive	1.59 (0.59, 4.24)	22 (57.9)	13 (46.4)	0.501
	Negative		16 (42.1)	15 (53.6)	
*VacA m2*	Positive	0.39 (0.14, 1.07)	13 (34.2)	16 (57.1)	0.109
	Negative		25 (65.8)	12 (42.9)	
*VacA s1*	Positive	2.90 (1.05, 8.06)	22 (57.9)	9 (32.1)	0.068
	Negative		16 (42.1)	19 (67.9)	
*VacA s2*	Positive	0.42 (0.16, 1.15)	15 (39.5)	17 (60.7)	0.145
	Negative		23 (60.5)	11 (39.3)	
*VacA s*-*^a^m1*	Positive	0.23 (0.02, 2.29)	1 (2.6)	3 (10.7)	0.304
	Negative		37 (97.4)	25 (89.3)	
*VacA s-m2*	Positive	–	1 (2.6)	0 (0.0)	1.000
	Negative		37 (97.4)	28 (100.0)	
*VacA s1m1*	Positive	2.68 (0.83, 8.65)	14 (36.8)	5 (17.9)	0.108
	Negative		24 (63.2)	23 (82.1)	
*VacA s1m*-*^b^*	Positive	–	2 (5.3)	0 (0.0)	0.504
	Negative		36 (94.7)	28 (100.0)	
*VacA s1m2*	Positive	1.12 (0.29, 4.43)	6 (15.9)	4 (14.3)	1.000
	Negative		32 (84.2)	24 (85.7)	
*VacA s2m1*	Positive	1.04 (0.29, 3.69)	7 (18.4)	5 (17.9)	1.000
	Negative		31 (81.6)	23 (82.1)	
*VacA s2m*-	Positive	–	2 (5.3)	0 (0.0)	0.504
	Negative		36 (94.7)	28 (100.0)	
*VacA s2m2*	Positive	0.23 (0.07, 0.78)	5 (13.2)	11 (39.3)	0.031
	Negative		33 (86.8)	17 (60.7)	

^a^The *s* genotype is unknown because it was not detected. ^b^The *m* genotype is unknown because it was not detected.

### Trends of *H. pylori* genotype expression over the study duration

**FIGURE 2:**
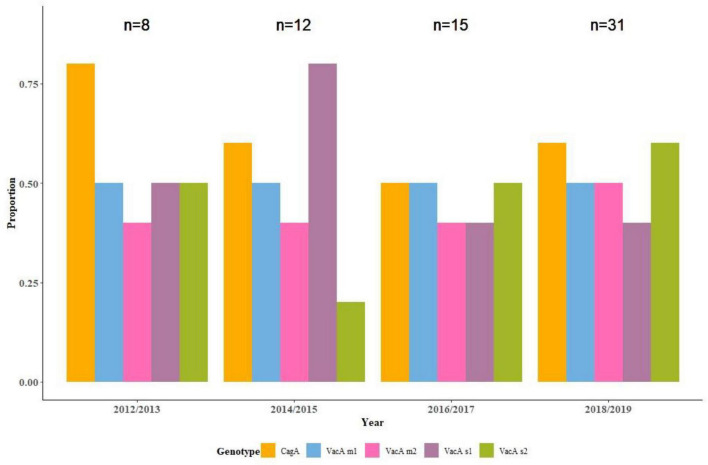
Trends in the distribution of *Helicobacter pylori* genotypes (*CagA*, *VacA s1*, *VacA s2*, *VacA m1*, and *VacA m2*) from 2012 to 2019.

Analysis of the trends of *H. pylori* genotypes over the 8 years, 2012 through 2019, was done using the 66 samples where molecular characterization was successful. This represented 23% of the entire sample size, and comprised 38 cases and 28 controls. Because of the small number in each year, two consecutive years were combined to create four time points. Genotypes with the greatest year-to-year variation as measured by percent coefficient of variation (%CV) were *VacA s1* and *VacA s2*, (%CV of 41 and 42, respectively). In contrast, *VacA m1* and *VacA m2* exhibited minimal variation (%CV of 4 and 11, respectively). The *CagA* genotype also showed marked variation (%CV 19). The trends are visualized in Figure 2.

## Discussion

Gastric cancer is the fifth most common malignancy, and the fourth leading cause of cancer-related deaths worldwide (5). On average, only 1–3% occur as part of a hereditary syndrome, with the greater majority of reported cases found to be sporadic, in which *H. pylori* infection has been established as the most important risk factor. As a strategy of mitigating gastric cancer risk therefore, both developed and low-and-middle income countries (LMIC) have instituted various *H. pylori* eradication programs. However, in spite of this fact, the latest GLOBOCAN report of 2020, evidenced an upsurge in gastric cancer cases, 70% of which were attributed to developing countries (5). This current study sought to evaluate the association of this bacterium and its virulence factors *CagA* and *VacA*, with gastric adenocarcinoma, and to describe the *H. pylori* genotype distribution in Kenya.

Similar to the global pattern, the rate of gastric cancer was higher in males than in females (ratio = 1.5:1). The median age at diagnosis was 61 years, and so, in contrast to previous studies, this study demonstrated a higher frequency in the elderly, rather than the previously reported third and fourth decade in African populations (6). Nevertheless, a significant proportion (11%), of gastric cancer occurred between the third and fourth decades, with the youngest patient being 21 years. These observations may be attributed to host and environmental risk factors, not evaluated in this study.

Concordant with the results of various gastric adenocarcinoma focused studies, intestinal variant was the most common (61.5%) followed by diffuse and mixed types (25.9 and 7.7%, respectively). Interestingly, however, these findings differed with a recently published study on gastric cancer in Kenya, where the authors found diffuse-type gastric cancer to be more common (15).

A total of 32% of the cases were found to be positive for the *H. pylori*, which was in contrast to a previously conducted local study in 2010, that demonstrated a prevalence of 0.9% (10). Further to this, more than half of the cases lacked a specified tumor site, 16.8% were from the GEJ and only 18.9% from the antrum. Given that the highest association with *H. pylori* is with antral carcinomas, it is possible that the low yield in this study, was due to the few numbers of antral biopsies. Other reasons could be low efficiency for recovery of bacterial DNA from FFPE, and also the “hit-and-run mechanism,” in which the pro-oncogenic actions of *H. pylori* virulence factors are taken over by a series of genetic alterations occurring in cancer-predisposed cells, during long-standing infection (16).

Overall, majority of the cases were poorly differentiated, and further, 60.9% of those positive for *H. pylori* exhibited poor differentiation. This finding was consistent with the concept that malignancies are generally diagnosed at a higher grade in LMIC; and this can be attributed to the absence of screening methods and barriers within the local health system, resulting in late diagnoses.

*VacA* is considered to be universal to all strains of *H. pylori*; and in brief, it works by binding to the surface of cells and inducing apoptosis, as well as inhibiting the proliferation and immune response of T lymphocytes (17). The “*s*” region corresponds to sequence differences within the terminal signal peptide, and the terminal end of the secreted toxin. The *s2* genotype, in comparison to *s1*, has an impaired ability to form channels in lipid bilayers, and so this allele generally has a reduced capacity to form vacuoles in mammalian cells (18). Therefore, the *s1* genotype is associated with increased severity of disease, compared to *s2*. The “*m*” region of diversity has variable vacuolating activity, which is largely dependent on the type of cell (18). Both alleles, however, have the capacity for vacuolating activity. In agreement with previous studies (9), the current study demonstrated that in comparison with the benign controls, *H. pylori* in the gastric adenocarcinoma cases were more likely *VacA m1* and *VacA s1* genotypes, (OR 1.59 and 2.90, respectively). As such, patients infected by *H. pylori* encoding for the virulent allelic combination *VacA s1m1*, although with wide CI (0.83–8.65), were almost three times more likely to be associated with cancer, than those infected by *H. pylori* of other genotypes. Conversely, the allelic combination of *VacA s2m2*, was almost four times less likely to be associated with cancer. In disagreement with the systematic review and meta-analysis by Pormohammad et al. however, *CagA* genotype in the current study was not associated with a higher risk of gastric cancer development, with an OR of less than one. One of the reasons for this finding, could be that there is low prevalence of the *CagPAI* region, known to enhance its virulence (19), in Kenyan strains of *H. pylori*.

The current study, focusing on *H. pylori* and gastric cancer revealed a divergent risk categorization for *H. pylori* genotypes: *VacA s1m1* with increased risk, and *VacA s2m2* with reduced risk of gastric cancer. Furthermore, over the 8-year study period (2012–2019), there has been an overall increase of *H. pylori* and in the expression of all its genotypes. The genotypes with the greatest year-to-year variation were *VacA s1* and *VacA s2*, (%CV of 41 and 42, respectively), in contrast with, *VacA m1* and *VacA m2*, which exhibited minimal variation (%CV of 4 and 11, respectively). The *CagA* genotype also showed marked variation (%CV 19) over the study duration. These findings would suggest that the evaluation of *VacA s1m1* and other genotypes, as opposed to the mere presence of *H. pylori*, can be used, not only to stratify patients at a higher risk of gastric malignancy, but also for epidemiologic studies.

The study utilized formalin fixed and paraffin embedded tissue blocks as both case and control samples; and despite this method allowing for the preservation of tissue architecture, which in turn allowed for acceptable histologic analyses, formalin is known to cause crosslinking of proteins and nucleic acids, as well as random breakages in the nucleotide sequences. This in turn could have resulted in false negative results in the molecular analyses of *H. pylori* and its genotypes, as well as failure to detect the expression of some genotypes (see Table 3).

In conclusion, the findings of this study thus put forward, that further assessment of the specific genes encoded by *H. pylori* isolates, in chronically infected persons, can aid in stratifying those at increased risk of development of gastric adenocarcinoma. The low prevalence of intestinal metaplasia and atrophic gastritis also highlights the need for further studies into host *H. pylori* interaction and gastric carcinogenesis in African populations.

## Data availability statement

The original contributions presented in this study are included in this article/Supplementary material, further inquiries can be directed to the corresponding author.

## Author contributions

PN and AN conceived and designed the study, collected, compiled and analyzed the data, and wrote the final manuscript. ZM and GR participated in the conception and design of the study, providing critical feedback that helped shape the research. YY and ET provided support in the form of training and materials required for the genotype analysis. All authors contributed to the article and approved the submitted version.

## Footnotes

1
https://ncbi.nlm.nih.gov

2
https://blast.ncbi.nlm.nih.gov
